# Supplementary material for: Papanicolaou stain unmixing for RGB image using weighted nucleus sparsity and total variation regularization
Source: Med Biol Eng Comput. 2025 Dec 16;64(3):911–29. doi: 10.1007/s11517-025-03490-z (PMC13061805; doi:10.1007/s11517-025-03490-z)
Supplement: Supplementary file 1 — Supplementary Material 1 [file 11517_2025_3490_MOESM1_ESM.docx]

Papanicolaou Stain Unmixing for RGB Image Using Weighted Nucleus Sparsity and Total Variation Regularization

Here, we detail the optimization algorithm of the proposed method introduced in Section 3. Given Prob. (16), we can reformulate it into the following formulation:

| $\min_{\mathbf{U,}\mathbf{V}_{\mathbf{1}}\mathbf{,}\mathbf{V}_{\mathbf{2}}\mathbf{,}\mathbf{V}_{\mathbf{3}}\mathbf{,}\mathbf{V}_{\mathbf{4}}\boldsymbol{,}\mathbf{V}_{\mathbf{5}}} \frac{1}{2}\left\Vert\mathbf{V}_{1}-\mathbf{Y} \right\Vert_{F}^{2}+\lambda\left\Vert\mathbf{W}\odot\mathbf{V}_{2} \right\Vert_{1,1}+\lambda_{TV}\left\Vert\mathbf{V}_{4} \right\Vert_{1,1}+\iota_{R+}\left( \mathbf{V}_{5} \right)$  $s.t.: \mathbf{V}_{1}=\mathbf{AX}, \mathbf{V}_{2}=\mathbf{X}, \mathbf{V}_{3}=\mathbf{X}, \mathbf{V}_{4}=\mathbf{H}\mathbf{V}_{3}, \mathbf{V}_{5}=\mathbf{X}$ | (S1) |
| --- | --- |

By letting

| $\mathbf{V}\equiv\left( \mathbf{V}_{1}\mathbf{,}\mathbf{V}_{2}\mathbf{,}\mathbf{V}_{3}\mathbf{,}\mathbf{V}_{4}\boldsymbol{,}\mathbf{V}_{5} \right)$,  $g\left( \mathbf{V} \right)\equiv\frac{1}{2}\left\Vert\mathbf{V}_{1}-\mathbf{Y} \right\Vert_{F}^{2}+\lambda\left\Vert\mathbf{W}\odot\mathbf{V}_{2} \right\Vert_{1,1}+\lambda_{TV}\left\Vert\mathbf{V}_{4} \right\Vert_{1,1}+\iota_{R+}\left( \mathbf{V}_{5} \right)$  $\mathbf{G=}\left[ \begin{matrix} \begin{matrix} \mathbf{A} \\ \mathbf{I} \end{matrix} \\ \mathbf{I} \\ \begin{matrix} \mathbf{0} \\ \mathbf{I} \end{matrix} \end{matrix} \right]\mathbf{, B=}\left[ \begin{matrix} \begin{matrix} \begin{matrix} \begin{matrix} \mathbf{-I} & \mathbf{0} \end{matrix} & \mathbf{0} & \begin{matrix} \mathbf{0} & \mathbf{0} \end{matrix} \end{matrix} \\ \begin{matrix} \begin{matrix} \mathbf{0} & \mathbf{-I} \end{matrix} & \mathbf{0} & \begin{matrix} \mathbf{0} & \mathbf{0} \end{matrix} \end{matrix} \end{matrix} \\ \begin{matrix} \begin{matrix} \mathbf{0} & \mathbf{0} \end{matrix} & \mathbf{-I} & \begin{matrix} \mathbf{0} & \mathbf{0} \end{matrix} \end{matrix} \\ \begin{matrix} \begin{matrix} \begin{matrix} \mathbf{0} & \mathbf{0} \end{matrix} & \mathbf{H} & \begin{matrix} \mathbf{-I} & \mathbf{0} \end{matrix} \end{matrix} \\ \begin{matrix} \begin{matrix} \mathbf{0} & \mathbf{0} \end{matrix} & \mathbf{0} & \begin{matrix} \mathbf{0} & \mathbf{-I} \end{matrix} \end{matrix} \end{matrix} \end{matrix} \right]$, | (S2) |
| --- | --- |

Prob. (S1) is reduced as follows:

| $\min_{\mathbf{U,V}} g\left( \mathbf{V} \right) s.t. \mathbf{GX+BV}=\mathbf{0}$. | (S3) |
| --- | --- |

The pseudocode solving Prob. (S3) is shown in Algorithm 1, where

| $L\left( \mathbf{X,V,D} \right)\equiv g\left( \boldsymbol{V} \right)+\frac{\mu}{2}\left\Vert\mathbf{GX+BV-D} \right\Vert_{F}^{2}$, | (S4) |
| --- | --- |

is the augmented Lagrangian for Prob. (S1). The constant $\mu$ is positive, and $\mathbf{D}$ denotes the Lagrange multipliers associated with the constraint $\mathbf{GX+BV}=\boldsymbol{0}$.

To show the optimization details we expand the augmented Lagrangian introduced in (S4):

| $L\left( \mathbf{X,}\mathbf{V}_{1}\mathbf{,}\mathbf{V}_{2}\mathbf{,}\mathbf{V}_{3}\mathbf{,}\mathbf{V}_{4}\mathbf{,}\mathbf{V}_{5}\mathbf{,}\mathbf{D}_{1}\mathbf{,}\mathbf{D}_{2}\mathbf{,}\mathbf{D}_{3}\mathbf{,}\mathbf{D}_{4}\mathbf{,}\mathbf{D}_{5} \right)$  $=\frac{1}{2}\left\Vert\mathbf{V}_{1}-\mathbf{Y} \right\Vert_{F}^{2}+\lambda\left\Vert\mathbf{W}\odot\mathbf{V}_{2} \right\Vert_{1,1}+\lambda_{TV}\left\Vert\mathbf{V}_{4} \right\Vert_{1,1}+\iota_{R+}\left( \mathbf{V}_{5} \right)+\frac{\mu}{2}\left\Vert\mathbf{AX-}\mathbf{V}_{1}\mathbf{-}\mathbf{D}_{1} \right\Vert_{F}^{2}$  $+\frac{\mu}{2}\left\Vert\mathbf{X-}\mathbf{V}_{2}\mathbf{-}\mathbf{D}_{2} \right\Vert_{F}^{2}+\frac{\mu}{2}\left\Vert\mathbf{X-}\mathbf{V}_{3}\mathbf{-}\mathbf{D}_{3} \right\Vert_{F}^{2}+\frac{\mu}{2}\left\Vert\mathbf{HV}_{3}\mathbf{-}\mathbf{V}_{4}\mathbf{-}\mathbf{D}_{4} \right\Vert_{F}^{2}+\frac{\mu}{2}\left\Vert\mathbf{X-}\mathbf{V}_{5}\mathbf{-}\mathbf{D}_{5} \right\Vert_{F}^{2}$ | (S5) |
| --- | --- |

The ADMM iteratively minimizes $L\left( \mathbf{X,V,D} \right)$ with respect to $\mathbf{X}$ and $\mathbf{V}$ followed by an update of $\mathbf{D}$ in the following steps. Thus, we have

| $\mathbf{X}^{(k+1)}=\left( \mathbf{A}\mathbf{A}^{T}+3\mathbf{I} \right)^{-1}(\mathbf{A}^{T}\left( \mathbf{V}_{1}^{\left( k \right)}+\mathbf{D}_{1}^{\left( k \right)} \right)+(\mathbf{V}_{2}^{\left( k \right)}+\mathbf{D}_{2}^{(k)})+(\mathbf{V}_{3}^{\left( k \right)}+\mathbf{D}_{3}^{\left( k \right)}) +(\mathbf{V}_{5}^{\left( k \right)}+\mathbf{D}_{5}^{\left( k \right)}))$ | (S6) |
| --- | --- |

To compute the optimization for $\mathbf{V}$, we have

| $\mathbf{V}_{1}^{\left( k+1 \right)}=\frac{1}{1+\mu}[\mathbf{Y}+\mu(\mathbf{A}\mathbf{X}^{\left( k+1 \right)}-\mathbf{D}_{1}^{\left( k \right)})]$  $\mathbf{V}_{2}^{\left( k+1 \right)}=\mathrm{soft}(\mathbf{X}^{\left( k+1 \right)}-\mathbf{D}_{2}^{\left( k \right)},\frac{\lambda}{\mu}\mathbf{W})$  $\mathbf{V}_{3}^{\left( k+1 \right)}=\left( \mathbf{H}^{T}\mathbf{H+}\mathbf{I} \right)^{-1}(\mathbf{X}^{\left( k+1 \right)}-\mathbf{D}_{3}^{\left( k \right)}+\mathbf{H}^{T}(\mathbf{V}_{4}^{\left( k \right)}+\mathbf{D}_{4}^{\left( k \right)}))$  $\mathbf{V}_{4}^{\left( k+1 \right)}=\mathrm{soft}(\mathbf{H}\mathbf{V}_{3}^{\left( k+1 \right)}-\mathbf{D}_{4}^{\left( k \right)},\frac{\lambda_{TV}}{\mu})$  $\mathbf{V}_{5}^{\left( k+1 \right)}=\max(\mathbf{X}^{\left( k+1 \right)}-\mathbf{D}_{5}^{\left( k \right)},0)$ | (S7) |
| --- | --- |

where $\mathrm{soft}(\cdot,\tau)$ denotes the component-wise application of the soft-threshold function $y\mapsto\mathrm{sign}(y)max\{\left| y \right|-\tau,0\}$. To compute the optimization for $\mathbf{D}$, we have

| $\begin{matrix} \begin{matrix} \mathbf{D}_{1}^{\left( k+1 \right)}=\mathbf{D}_{1}^{\left( k \right)}-\mathbf{A}\mathbf{X}^{\left( k+1 \right)}+\mathbf{V}_{1}^{\left( k+1 \right)} \\ \mathbf{D}_{2}^{\left( k+1 \right)}=\mathbf{D}_{2}^{\left( k \right)}-\mathbf{X}^{\left( k+1 \right)}+\mathbf{V}_{2}^{\left( k+1 \right)} \end{matrix} \\ \mathbf{D}_{3}^{\left( k+1 \right)}=\mathbf{D}_{3}^{\left( k \right)}-\mathbf{X}^{\left( k+1 \right)}+\mathbf{V}_{3}^{\left( k+1 \right)} \\ \begin{matrix} \mathbf{D}_{4}^{\left( k+1 \right)}=\mathbf{D}_{4}^{\left( k \right)}-\mathbf{H}\mathbf{V}_{3}^{\left( k+1 \right)}+\mathbf{V}_{4}^{\left( k+1 \right)} \\ \mathbf{D}_{5}^{\left( k+1 \right)}=\mathbf{D}_{5}^{\left( k \right)}-\mathbf{X}^{\left( k+1 \right)}+\mathbf{V}_{5}^{\left( k+1 \right)}. \end{matrix} \end{matrix}$ | (S8) |
| --- | --- |

Concerning computational complexity, let $L$ be the number of spectral channels, $r$ the number of dyes, and $N$ the number of pixels in an image. Each ADMM iteration costs $\mathcal{O((}Lr+r^{2})N)$ for linear algebra, plus $\mathcal{O(}rN)$ for proximal updates. With TV regularization enabled, the TV subproblem contributes $\mathcal{O(}rN\log N)$ per iteration. Hence, an iteration costs approximately $\mathcal{O(}N\log N)$. Memory scales approximately $\mathcal{O(}N)$.

| **Algorithm 1:** Pseudocode for solving Prob. (S1). |
| --- |
| 1: **Initialization:**  set $k=0$, choose $\mu>0,\mathbf{X}^{(0)}, \mathbf{V}^{(0)}, \mathbf{D}^{(0)}$  2: **repeat:**  3: $\mathbf{W}^{(k+1)}=\left[ \begin{matrix} \begin{matrix} \mathbf{0} \\ exp(-\mathbf{X}^{\left( k \right)}(2,:)) \end{matrix} \\ \begin{matrix} \mathbf{0} \\ \mathbf{0} \end{matrix} \end{matrix} \right]$  4: $\mathbf{X}^{(k+1)}⟵\mathrm{argmin}_{\mathbf{X}}L(\mathbf{X},\mathbf{V}^{\left( k \right)},\mathbf{D}^{(k)})$  5: $\mathbf{V}^{(k+1)}⟵\mathrm{argmin}_{\mathbf{V}}L(\mathbf{X}^{\left( k+1 \right)},\mathbf{V},\mathbf{D}^{(k)})$  6: $\mathbf{D}^{(k+1)}⟵\mathbf{D}^{(k)}-\mathbf{G}\mathbf{X}^{(k+1)}-\mathbf{B}\mathbf{V}^{(k+1)}$  7: **Update iteration:** $k⟵k+1$  8: **until** some stopping criterion is satisfied. |
