# Supplementary material for: Papanicolaou stain unmixing for RGB image using weighted nucleus sparsity and total variation regularization
Source: Med Biol Eng Comput. 2025 Dec 16;64(3):911–29. doi: 10.1007/s11517-025-03490-z (PMC13061805; doi:10.1007/s11517-025-03490-z)
Supplement: Supplementary file 2 — Supplementary Material 2 [file 11517_2025_3490_MOESM2_ESM.docx]

Papanicolaou Stain Unmixing for RGB Image Using Weighted Nucleus Sparsity and Total Variation Regularization

*Proposed stain unmixing method on different RGB images*

To evaluate whether the proposed method can adapt to different stain matrices and remain robust to color variations in RGB images, we preliminarily examined its robustness to variations in the staining matrix. The stain matrix may be influenced by different chemical recipes as well as the characteristics of RGB imaging systems. In this verification, we applied the proposed stain unmixing method to images acquired from two distinct imaging systems, MS-RGB and WSI-RGB, to examine its consistency across acquisition conditions.

First, stain matrices for unmixing were estimated from single-stain images captured by each imaging system (Fig. s1). As expected, noticeable differences were observed among the estimated stain matrices due to system-specific color variations.


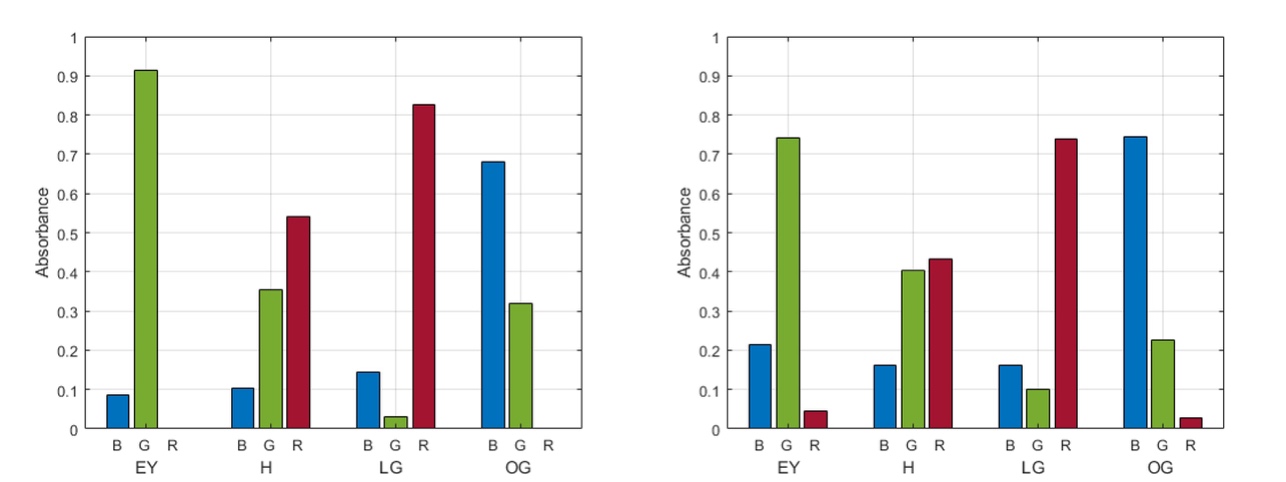


**Fig. s1** Normalized MS-RGB absorption coefficients (left), and WSI-RGB absorption coefficients (right) of Papanicolaou stain

Using these matrices, our proposed stain unmixing method was then applied to unmix Papanicolaou-stained RGB images from each system into individual dye channels. Subsequently, stain abundances were normalized based on the robust maximum abundance of each dye within the corresponding imaging system. Images from the two datasets were analyzed by comparing raw RGB intensities with the unmixed stain abundances (Fig. s2). Although the same specimen displayed different RGB intensities across imaging systems, the normalized stain abundances were nearly identical. These preliminary results support the effectiveness of the proposed method in adapting to different stain matrices and mitigating color variations introduced by imaging systems.


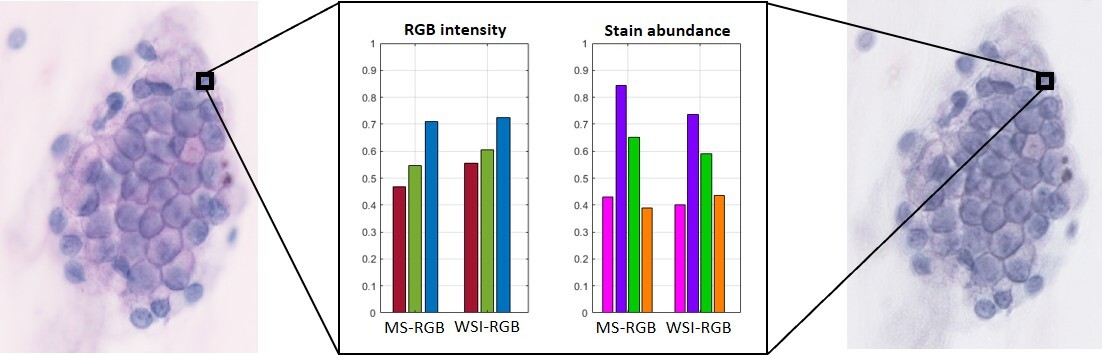


**Fig. s2** RGB intensities and stain abundances unmixed by the proposed method for the same ROI in the MS-RGB image (left) and the WSI-RGB image (right). Although the RGB intensities and stain matrices differ between the two image sets, the unmixed stain abundances are nearly identical

This experiment primarily addressed color variation induced by imaging systems, but not variation due to staining conditions. Because suitable publicly available Papanicolaou-stained datasets are limited, we are supplementing our dataset with images prepared under different staining conditions. In future work, we plan to conduct a more systematic and comprehensive investigation using multi-institutional Papanicolaou-stained datasets to fully evaluate the adaptability of the method to diverse sources of color variations.
